# Review of State Comprehensive Cancer Control Plans for Genomics Content

**Published:** 2005-03-15

**Authors:** Debra E Irwin, Erin Shaughnessy Zuiker, Tejinder Rakhra-Burris, Robert C Millikan

**Affiliations:** North Carolina Center for Genomics and Public Health, Department of Epidemiology, School of Public Health; North Carolina Center for Genomics and Public Health, School of Public Health, University of North Carolina at Chapel Hill, Chapel Hill, NC; North Carolina Center for Genomics and Public Health, School of Public Health, University of North Carolina at Chapel Hill, Chapel Hill, NC; Department of Epidemiology, School of Public Health, University of North Carolina at Chapel Hill, Chapel Hill, NC

## Abstract

**Introduction:**

The goals of this study were to determine U.S. states with Comprehensive Cancer Control plans that include genomics in some capacity and to review successes with and barriers to implementation of genomics-related cancer control initiatives.

**Methods:**

This study was conducted in two phases. Phase one included a content analysis of written state Comprehensive Cancer Control plans (n = 30) for terms related to genomics, or "genomic components" (n = 18). The second phase involved telephone interviews with the Comprehensive Cancer Control plan coordinators in states with plans that contained genomic components (n = 16). The interview was designed to gather more detailed information about the genomics-related initiatives within the state's Comprehensive Cancer Control plan and the successes with and barriers to plan implementation, as defined by each state.

**Results:**

Eighteen of the 30 Comprehensive Cancer Control plans analyzed contained genomics components. We noted a large variability among these 18 plans in the types of genomics components included. Nine (56%) of the 16 states interviewed had begun to implement the genomics components in their plan. Most states emphasized educating health care providers and the public about the role of genomics in cancer control. Many states consider awareness of family history to be an important aspect of their Comprehensive Cancer Control plan. Approximately 67% of states with family history components in their plans had begun to implement these goals. Virtually all states reported they would benefit from additional training in cancer genetics and general public health genomics.

**Conclusion:**

The number of states incorporating genomics into their Comprehensive Cancer Control plans is increasing. Family history is a public health application of genomics that could be implemented more fully into Comprehensive Cancer Control plans.

## Introduction

Comprehensive Cancer Control (CCC) is an emerging public health model that seeks to bring together public and private stakeholders to efficiently use limited resources to reduce the burden of cancer. The CCC program allows states and territories to facilitate their own partnerships to address their unique cancer burdens. CCC results in many benefits, including increased efficiency for delivering public health messages and services to the public. The Centers for Disease Control and Prevention's (CDC's) National CCC Program (NCCCP) is a resource for supporting CCC efforts. Since 1998, the number of programs participating in NCCCP has grown from six to 61 (1). With this support, state, tribal, and territorial health agencies continue to establish broad-based CCC coalitions, to assess the burden of cancer, to determine priorities for cancer prevention and control, and to develop and implement CCC plans. State planners play a large and important role in CCC programs as the cancer burden increases for the population and advances in cancer genomics continue to challenge public health specialists.

For public health purposes, genetics may be defined as the "study of single gene hereditability," whereas genomics is the study of functions and interactions of all the genes in the genome, including their interactions with environmental factors (2). It is estimated that 5% to 10% of cancer is caused by autosomal dominant inherited genetic changes, such as *BRCA1* and *BRCA2* mutations in breast and ovarian cancer (3). Family history of cancer in a first-degree relative has been shown to confer an increased cancer risk (e.g., the relative risk of breast cancer conferred by a first-degree relative with breast cancer is 2.1) (4). Individuals who may have a genetic susceptibility because of cancer in their family can be distinguished from individuals in the general population by the relatively straightforward process of taking a family history. The American Society of Clinical Oncology supports integrating cancer risk assessment and management, including genetic testing for cancer predisposition genes, into the practice of oncology and preventive medicine (5).

The states are committed to reducing the burden of cancer among their populations, and the emerging contribution of genetics and genomics to the field of cancer control cannot be ignored. The goals of this study were to determine U.S. states that include genomics in some capacity in their CCC plans and to review the successes with and barriers to implementation of these genomics-related state cancer control initiatives.

## Methods

This study was conducted in two phases. The first phase was a content review of written state cancer control plans. In collaboration with the CDC, the North Carolina Center for Genomics and Public Health (NCCGPH) identified state CCC plans funded by the CDC from 1997 to 2004. Each plan was searched for the words "genetics," "genomics," "genes," "family history," "DNA," "first-degree relative," and "heritability." The search terms were identical to those chosen by the CDC for an earlier content analysis. These search terms were used to create a comprehensive list of potential genomics-related topics found within the state plans. Throughout this document, these terms will be referred to as "genomic components."

Several themes from among the genomic components were detected across plans, and these were tabulated. A report was written summarizing overall themes, supplying standardized definitions of genomic components, and detailing genomic components found in each state's CCC plan. 

Once the written CCC plans were reviewed, five topic areas were identified as areas needing more information to provide a more complete picture of the genomic components within the plans. The five topic areas were 1) the CCC plan writing process; 2) successes with and barriers to implementation; 3) general public and health care provider education programs that may have been implemented; 4) priority of genomics in the state health department; and 5) additional partnerships, training, and technical assistance that would be useful for CCC coordinators, coalitions, and state cancer control planners. NCCGPH staff, in consultation with the CDC, developed a telephone interview to gather additional information on these topics. The Institutional Review Board of the University of North Carolina approved the interview component of the study.

The second phase of the study involved telephone interviews with the CCC plan coordinators in states with genomic components in their cancer control plans. Sixteen of the 18 states agreed to be interviewed. The summary report from the content review of the written CCC plans (Phase 1) was sent to all 16 of the state CCC coordinators for their review prior to their scheduled interview. At the beginning of the interview, the CCC coordinators were asked to verify that the summary of genomic components for their state was accurate and complete. No changes or additions were made by any of the states interviewed. All interviews were audiotaped and transcribed, and copies of the transcriptions were sent back to each state for quality control purposes and their final approval.

The interview used a semistructured questionnaire (Appendix) and gathered information about only the genomic components within the CCC plan. (The other elements of the CCC plans were not discussed in the interview.) The questions addressed the five topics listed above. Each state was allowed to determine if implementation of the "genomic components" had begun based on the context of their state plan. In addition, standardized definitions of "success and barriers" were not imposed; each state was allowed to determine success based on its plan and goals.

## Results

### Summary of written Comprehensive Cancer Control plans

Of the 30 CCC plans analyzed, 18 contained genomic components. Among these 18 plans, we found large variability in the types of genomic components included. Table 1 summarizes the frequency of the main themes among the CCC plans. Most states used the terms "genetics" or "family history," while only one state referred to "genomics" in the written CCC plan. Half of the states intended to monitor advances in the cancer genomics field by publishing new information through their in-house newsletters, convening advisory panels, working with statewide experts in the field, and providing professional educational programs. Slightly more than one quarter of the states discussed gene–environment interactions in any context. Gene–environment interactions were discussed under a variety of topics; for example, variations seen in incidence rates among racial and ethnic groups for certain cancers, genetic research studies on various nutrients, and the relationship between inherited susceptibility and environmental factors for some cancers.

One theme, education, consistently presented itself in two forms: 1) increasing awareness about genomics among health care providers, and 2) providing education about genomics and its role in cancer control to patients and the general public. Approximately 44% of the CCC plans targeted education of health care providers and the public to promote early screening for those individuals identified at higher risk of cancer based on family history (data not shown). Also, one third of plans (33%) mentioned training health care professionals in the use of cancer risk assessment, including the use of family history tools.

### Summary of interviews

Nine out of the 16 states interviewed had begun implementation of the genomic components of their plan at the time of our interview (Table 2). All of these states were funded through implementation-type grants. States that reported initiation of implementation projects did so largely through educational forums or seminars, presentations at professional meetings, publication and distribution of fact sheets on specific cancers, and public service announcements (PSAs) that included issues of family history. Only two states reported that genomics was somewhat not a priority within their state health department (Table 2). Many states (43.7%) reported implementing education efforts aimed at health care providers, and 25% of the states reported providing some form of public education about genomics. Educational efforts have been accomplished mainly through holding open meetings and seminars, attending public health fairs, publishing fact sheets, issuing PSAs, and developing Web sites (Table 2).

Six states (data not shown) discussed implementation of their objectives to educate the public about family history. Initiatives included developing fact sheets, brochures, and Web sites discussing individual cancers and the role that family history plays as an important risk factor to consider when assessing cancer risk and the need for early screening. Some of these states held forums for health care professionals to discuss the importance of family history as a tool in assessing cancer risk. One state has convened a panel and developed a pilot to use the state cancer registry to help identify families at high risk for cancer development.

The primary reasons cited for successful implementation of genomic components within the state CCC plans were the following: 1) establishing strong partnerships within the state; 2) obtaining additional funding for implementation; and 3) making genomics a high priority within the state health department (Table 3). The types of partnerships varied and included private industry, major medical centers within the state, public research institutions, and universities. Many of the advisory committees had members who convened an array of partners within the state. Funding was obtained from national and local organizations, private industry, academic institutions, and other public resources. As expected, lack of additional funding and competing priorities were the major reasons cited as barriers to successful implementation (Table 3).

Virtually all of the states interviewed indicated that they would welcome additional training (n = 14) and/or technical assistance (n = 6) in genomics. States requesting additional training preferred some level of interpersonal interaction (100%), with the essential component being a live person to field questions, whether that be via phone, video, the Internet, or a face-to-face training session.

A basic public health genomics course was requested by 12 states, with topics including 1) the definition of genetics vs genomics; 2) risk assessment and family history issues; 3) proteomics; and 4) gene–environment interaction (Table 4). Three states requested training in the ethical, legal, and social issues (ELSI) of cancer genomics (Table 4). And six states requested a template or "how to" guide for implementing genomics issues into cancer control (Table 4). In addition to training requests, six states requested technical assistance; five of these six states requested program planning, implementation, and evaluation services (Table 4).

## Discussion

As expected, we noted a great deal of inconsistency in both the overall content and the level of detail within specific action plans. It is important to note that the dates and coverage of the plans range from 1997 to 2008. The more recently published plans have more extensive genomics content. For example, early plans (published in 1997 or 1998) do not include a section on breast cancer and genetic testing for susceptibility. The primary genomic components within these earlier plans are related to family history as a cancer risk factor. Conversely, plans published after 2000 provide more information on genetic testing for inherited breast cancer susceptibility (*BRCA1 *and *BRCA2* genes) as well as brief discussions of familial risk assessment.

Individuals involved in writing the plans and the process that each state underwent to write the plans may also have contributed to the variability in genomic components seen among the CCC plans. Some of the individuals interviewed for this review were not on staff at the time the plans were written and could only provide limited information regarding the process. However, all of the states used a collaborative writing process, involving several individuals with varying expertise who came together to draft the plans. In addition, the individuals interviewed may not be aware of all the programs that are ongoing within their state, so these results may reflect a subset of genomics-related activities within the state health department.

Nine of the 16 states we interviewed had begun to implement genomics-related projects within their CCC plan. Implementation was not strictly defined for the states, but instead states were allowed to determine whether or not implementation had begun based on the context of their state plan. The states were given the opportunity to define "successful" for the context of their program. Similarly, a standardized definition of "success and barriers" was not imposed. Hence, there is most likely variability in the interpretation of these terms.

Some of the state implementation projects were specifically designed to address genomics-related goals and objectives within the CCC plan, while other states have chosen to implement broader programs to address genomics-related CCC components as well as other CCC plan goals. These implementation projects varied greatly among the states and included such activities as creating Web sites and fact sheets and developing innovative public and health care provider educational programs. For example, one state trained local barbers in an ethnic community as "lay educators" to promote prostate cancer awareness, including risk from family history. Using the barbershop as a gathering place, the lay educators provided literature and information to clientele about the importance of early screening and family history risk. A video was also created to play in the barbershop to provide more information about prostate cancer.

It is interesting to note that only one of the plans reviewed actually used the term "genomics"; the others used the term "genetics." The one plan that used the term "genomics" was recently updated, reflecting the fact that genomics is a relatively new concept. The understanding of and use of this term may not yet be fully incorporated into public health practice. In addition, because "genomics" is new terminology, some states may have chosen not to use the term in order to make their plans more reader friendly.

Education is a theme that consistently presented itself in two forms: 1) increasing awareness about genomics among legislators and health care providers, and 2) providing education about genomics and its role in cancer control to patients, providers, and the general public. All of the plans discuss educating the public about early screening and prevention, specifically for breast, ovarian, colon, and prostate cancers. Most plans discuss the emerging field of cancer genetics, and all of the plans mention the need to monitor ongoing research and advances within the field. Educational programs were implemented as part of ongoing seminars or as stand-alone events, including fairs, athletic events, and social hours. Their success was reported as being largely dependent on aspects of their presentation (appropriate topics, dynamic speakers), timing (appropriate length for the event) and successful advertising.

The emphasis placed on raising awareness and educating health care providers and the public may reflect the time at which the plans were written, which was still early in the process of integrating genetics into public health cancer control efforts. This result seems appropriate given the early stages of the field of cancer genomics and available public health applications at the time of publication. Also, there were few commercially available tests for cancer genes that showed a significant public health benefit at the time these state plans were developed.

Using family history as a risk assessment tool is an important component within cancer genetics and one of the most amenable public health applications of genomics at this time (5-8). Genetic testing should be offered when an individual has a family history suggesting a genetic cancer susceptibility condition (5). Several states simply mention that family history is a risk factor for specific cancers, such as breast, colon, and prostate cancer. Other states dedicate entire sections to family history and call for educating providers about its use in cancer risk assessment and training them to detect patterns of inheritance and differentiating hereditary syndromes. Family history is a public health application of genomics that could be implemented more fully into CCC plans through awareness and education efforts. 

In its 2003 annual report, the CDC identified the premature commercialization of genetic tests — before safety, efficacy, and cost-effectiveness had been established — as one of the key issues in genetic testing (9). The year 2003 brought the first direct-to-consumer advertising for an inherited breast and ovarian cancer susceptibility genetic test (*BRCA1* and *BRCA2*). Given this recent development, it is not surprising that none of the reviewed plans discussed the impact of commercialization of genetic testing and direct-to-consumer marketing for genetic susceptibility tests. As technology advances and more tests are available to the public, there will likely be an increase in this type of marketing activity by commercial entities. This development highlights the increasing importance of providing education about informed uses of genetic testing as it relates to cancer.

Some states (approximately one third) identified reasons for success in implementation of the genomic components of their state plans. Predominantly, these included securing adequate funding, developing excellent partnerships, and having genetics deemed a high priority within the state health department. Two states noted that the resources within the states, such as having staff dedicated to public health genomics, increased the likelihood of successful implementation. As expected, the primary barriers to successful implementation of the genomic components were lack of funding and competing priorities. Almost 90% of states (14/16) interviewed were interested in obtaining additional genomics-related training and/or technical assistance.

In summary, the number of states incorporating genomic components into their CCC plans is increasing. These states are beginning to implement these objectives. Periodic reviews of the successes and barriers related to implementation of genomic components should continue so as to document progress and share the lessons learned from these experiences.

## Figures and Tables

**Table 1:** Summary of Genomics-related Themes Among 18 State Comprehensive Cancer Control (CCC) Plans^a^

**Theme**	**No. Plans (%)**
Used term “genomics”^a^	1 (5.6)
Used term “genetics”^b^	17 (94.4)
Discussed “family history”^c^	17 (94.4)
Discussed training health care professionals to use family history for cancer risk assessment^d^	6 (33.3)
Discussed providing general education for health care providers^e^	10 (55.6)
Discussed providing general education for public^f^	8 (44.4)
Discussed monitoring ongoing research and advances^g^	9 (50.0)
Discussed gene–environment interactions^h^	5 (27.8)

a Plan mentioned “genomics” in any context.

b Plan used the term “genetic” or “genetics” in any context.

c Plan discussed “family history” as related to cancer, most often stated as a risk factor for cancer.

d Plan specifically discussed training any health care providers to use family history as a tool for assessing risk.

e Plan mentioned providing some form of education for any type of health care providers about cancer genetics.

f Plan mentioned providing some form of general education for the public about cancer genetics, genetic predisposition for cancer, or the importance of genetic markers.

g Plan mentioned monitoring ongoing research/advances in cancer genetics, the field of genetics, or ethical, legal, and social implications of cancer genetics.

h Plan mentioned gene–environment interactions as related to cancer.

**Table 2:** Profile of 16 State Comprehensive Cancer Control (CCC) Plans with Genomics Components

**CCC Plan**	**No. Plans (%)**

**Type of Grant**	

Planning	3 (18.8)
Implementation	13 (81.2)
Have begun to put genomic components of plan into action	9 (56.2)
Have begun to draft a new CCC plan	9 (56.2)
Provided general public education about genomics	4 (25.0)
Provided health care provider education about genomics	7 (43.8)

**Perceived priority level of genomics within the state health department**	

High priority	5 (31.2)
Somewhat a priority	9 (56.2)
Somewhat not a priority	2 (12.5)
Not a priority	0 (0)

**Table 3:** Identified Successes With and Barriers to Implementation of State Comprehensive Cancer Control (CCC) Plans^a^

**Successes**	**No. Plans (%)**	**Barriers**	**No. Plans (%)**
Excellent partnerships and strong research/medical community in state	5 (31.2)	Lack sufficient funding	6 (37.5)
High priority	3 (18.8)	Misperceptions/ misinformation among public about genomics	4 (25.0)
Genetic counselor on staff	2 (12.5)	Lack of sufficient staff/leadership	3 (18.8)
Additional funding sources	2 (12.5)	Low priority	3 (18.8)
Provide continuing credits for professionals (i.e., CMEs, CEUs)	2 (12.5)	Time constraints	2 (12.5)
		Lack model/template to apply genetics	2 (12.5)

a A state could report more than one success and/or barrier; percentages are based on 16 plans.

**Table 4:** Training and Technical Assistance Needs in Genomics of 16 States with Genomics Components of Comprehensive Cancer Control Plan

	**No. (%)**

**States requesting training**	**14 (87.5)**

**Preferred topics^a^**	
Basic public health genomics concepts	11 (68.8)
Genomics vs genetics defined	4 (25.0)
ELSI^b^	3 (18.8)
“How to” guide for implementation of genomics components	6 (37.5)

**States requesting technical assistance**	**6 (37.5)**

Program planning, implementation, and evaluation services	5 (31.3)

a States may have responded with more than one training topic; percentages are based on 16 state plans.

b ELSI = ethical, legal, and social implications.

## Appendix: Questions for State CCC Plan Interviews

Have you had a chance to read through the fact sheet we mailed to you?

_______no (read through and review contents of the fact sheet)

_______yes

Do you have any questions at this point?

_____no

_____yes (resolve/answer questions)

Do you agree to participate in this study?

_____no (stop interview)

_____yes (continue with interview)

Thank you. On behalf of NCCGPH, we are grateful that you have agreed to participate in this study of genomics content in your state’s Comprehensive Cancer Control plan. This interview should take about 45 minutes of your time. I would like to remind you that the following questions refer to the genomics components of your CCC plan and not to other activities. Upon completion of the interview, a written summary of your interview will be sent to you for verification and approval.

Do I have your permission to audiotape this interview to facilitate accurate recording of your answers? 

_____no (do not turn on the tape recorder)

_____yes

Let’s begin.

1. Have you had a chance to review the materials we sent you about your cancer plan? 
  _____no (reschedule the interview)
  _____yes
  If yes, are there any sections in your plan relating to genetics/genomics that were missed or that are inaccurate in our review? If so, please describe these sections.
  Do you have any additional comments regarding the review?
2. What type of grant does your state have for CCC development?
  _____planning
  _____implementation
  _____other (specify ______________________)
3. Who was involved in writing the genetics/genomics sections of your CCC plan?
  What is their background as it relates to genetics?
  How were these individuals selected?
4. How long did the writing process take for the genetic sections?
  What was the general process/procedure used to write the genetic sections?
5. To date, have you had the opportunity to implement the genetic components within your action plans?
  _____no (go to 6)
  _____yes
  If yes, what has been accomplished thus far? 
  If you have begun to implement the genetic components detailed in your state plan, how have you been successful in your implementation?  Please explain.
6. If you have begun to implement the genetic components detailed in your state plan, have you encountered any barriers?
  _____no (go to 7)
  _____yes
  If yes, please explain.
7. Have you provided any activities or programs to educate the public about genetics and cancer?
  _____no (go to 8)
  _____yes
  If yes, please list or describe these activities or programs.
  Using the following 4-point scale where 1 = received positively, 2 = somewhat positively, 3 = somewhat not positively, or 4 = not positively at all, how have the activities or programs been received? Received positively, somewhat positively, somewhat not positively, or not positively at all?  
  Why do you think these results were obtained?
8. Have you provided any activities or programs to educate health care providers about genetics and cancer?
  _____no (go to 9)
  _____yes
  If yes, please list or describe these activities or programs.
  On the same 4-point scale, how have these activities or programs been received: received positively, somewhat positively, somewhat not positively, or not positively at all?
  Why do you think these results were obtained?
9. Please rank genomics in terms of priority level for your state health department on the 4-point scale, where 1 = high priority, 2 = somewhat a priority, 3 = somewhat not a priority and 4 = not a priority. Is genomics a priority, somewhat of a priority, somewhat not a priority, or not a priority?
  Explain your answer. 
10. Have you begun the process of drafting a subsequent CCC plan?
  _____no (go to 11)
  _____yes
  If yes, has the genomics components changed?
  _____no
  _____yes
  _____not sure — plan not completed
  Why or why not?
  What is the planned date of issue for the new CCC plan?
  Is it an implementation grant?
  _____yes
  _____no (if no, specify what the type)
11. Does your state have genetic nondiscrimination legislation in place currently?
  _____no
  _____yes
  _____don’t know
12. As you move forward, what types of partnerships would be helpful to you in implementing the genomics components of your state plan?
13. Do you or your staff need additional training in genomics to assist in implementation of the genetic components of your state plan?
  _____no (go to 14)
  _____yes
  _____not sure
  If yes, what specific topics would you need training in to assist in implementation of the genetic components of your state plan?
  Which of the following training formats would you most prefer? 
  _____Interactive videoconference
  _____CD-ROM (interactive)
  _____Phone conference call
  _____Internet accessible 
  _____Standard videotape (not interactive)
14. Are you aware of the currently funded resources available to you through the three national genomic centers funded by the CDC? 
  _____yes (end interview)
  _____no
  _____not sure
  If not, interviewer should explain services. 
  Would these types of services be of use to you? 
  _____no (end interview)
  _____yes
  _____not sure
  What type of partnership with the genomic centers would be the most feasible? 
  Follow up with asking specifics that they would like or need help with.

Thank you very much for your time today.  Do you have any questions for me?  I’ll be sending you a summary of our interview for you to review and approve.  Where should I send this?

Record Address:

_______________________________________

Interviewer Signature

_______________________________________
